# Iodide transport: implications for health and disease

**DOI:** 10.1186/1687-9856-2014-8

**Published:** 2014-05-30

**Authors:** Liuska Pesce, Peter Kopp

**Affiliations:** 1 Stead Family Department of Pediatrics, Division of Pediatric Endocrinology and Diabetes, University of Iowa Carver School of Medicine, Iowa City, Iowa 52242, USA; 2 Department of Internal Medicine, Division of Endocrinology, Metabolism and Molecular Medicine, Feinberg School of Medicine, Northwestern University, Chicago, Illinois 60611, USA

**Keywords:** Iodide transport, Iodine, Thyroid, Thyroid hormones, Hypothyroidism, Hyperthyroidism, Thyroid cancer, Iodine deficiency, Radioactive iodine

## Abstract

Disorders of the thyroid gland are among the most common conditions diagnosed and managed by pediatric endocrinologists. Thyroid hormone synthesis depends on normal iodide transport and knowledge of its regulation is fundamental to understand the etiology and management of congenital and acquired thyroid conditions such as hypothyroidism and hyperthyroidism. The ability of the thyroid to concentrate iodine is also widely used as a tool for the diagnosis of thyroid diseases and in the management and follow up of the most common type of endocrine cancers: papillary and follicular thyroid cancer. More recently, the regulation of iodide transport has also been the center of attention to improve the management of poorly differentiated thyroid cancer. Iodine deficiency disorders (goiter, impaired mental development) due to insufficient nutritional intake remain a universal public health problem. Thyroid function can also be influenced by medications that contain iodide or interfere with iodide metabolism such as iodinated contrast agents, povidone, lithium and amiodarone. In addition, some environmental pollutants such as perchlorate, thiocyanate and nitrates may affect iodide transport. Furthermore, nuclear accidents increase the risk of developing thyroid cancer and the therapy used to prevent exposure to these isotopes relies on the ability of the thyroid to concentrate iodine. The array of disorders involving iodide transport affect individuals during the whole life span and, if undiagnosed or improperly managed, they can have a profound impact on growth, metabolism, cognitive development and quality of life.

## Introduction

Iodine, as its water-soluble iodide ion (I^−^), is the rate-limiting substrate for thyroid hormone synthesis. The availability of iodide depends on oral intake and the recommended daily allowances are summarized in Table 1. Iodide is absorbed in the stomach and duodenum and cleared by the kidney and the thyroid. Seventy to eighty percent of the iodine body content is located in the thyroid gland and thyroid hormone synthesis requires a series of regulated steps. Altered regulation or defects in any of these steps can affect thyroid hormone synthesis and secretion. Furthermore, the understanding of iodide transport is used in the diagnosis, prevention and treatment of thyroid disorders and knowledge about the mechanisms underlying iodide transport is now applied to treat advanced forms of thyroid cancer and non-thyroidal malignancies.

**Table 1:** **Recommendations for iodine intake by age and population group from the World Health Organization (WHO), UNICEF and ICCIDD**[1]

**Age or population group**	**Recommended daily allowance (μg)**
Pre-school children (0–59 months)	90
School children (6–12 years)	120
Adolescents and adults (above 12 years)	150
Pregnant and lactating women	250

### Iodine intake and absorption

Iodine, as iodide (I^−^), is available but not equally distributed in the environment. Most iodide is found in the oceans (sea water has 50 μg/L) and deficient soils are common in mountainous areas, regions that were glaciated and areas of frequent flooding; however, deficiency is also a problem in some coastal and island populations [2-5].

**Figure 1:**
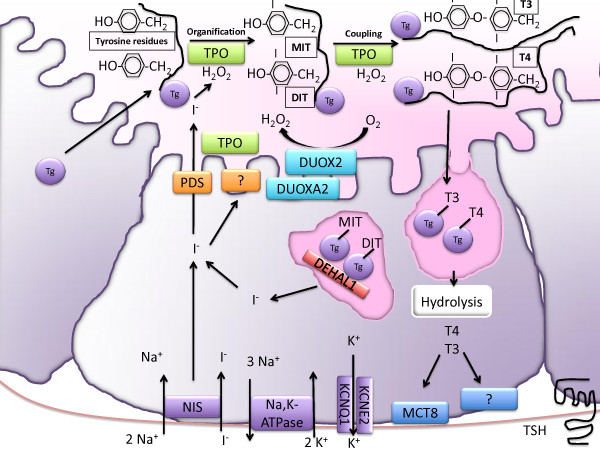
**Mechanisms of Iodide transport in thyroid follicular cells.** The first step in iodide uptake is mediated by the sodium-iodide symporter NIS, using the sodium gradient generated by the Na, K-ATPase. Active transport of potassium by the KCNE2/KCNQ1 potassium channel is also important, likely for maintaining the membrane potential of thyroid cells. At the apical membrane, pendrin and another yet unidentified transporter mediate iodide efflux. TPO, using H_2_O_2_ generated by the DUOX2/DUOXA system mediates the oxidation, organification and coupling reaction that result in the synthesis of the iodothyronines T4 and T3. Iodinated thyroglobulin is taken into the cell by micro- and macropinocytosis and digested in lysosomes. T4 and T3 are excreted via MCT8 and other transporters. The iodotyrosines MIT and DIT are dehalogenated by DEHAL1 and the released iodide is recycled. Purple boxes represent steps in basal iodide uptake. Orange boxes represent apical iodide uptake, oxidation, organification and coupling are mediated by TPO, represented in green boxes. The generation of H_2_O_2_ is represented in aqua. The recycling of iodide after digestion of iodinated thyroglobulin is represented in the red box. The secretion of thyroid hormones at the basolateral membrane is shown in the blue boxes.

Plants grown in iodine deficient soils have as low as 10 μg/kg of dry weight, while plants grown in iodine rich soils have a concentration of 1 mg/kg. Overall, the natural iodine content of many foods and beverages is low (3–80 μg per serving), while foods from marine origin have a higher content. However, sea salt has negligible amounts, as iodide in seawater is sublimated into the atmosphere as volatile organic iodine [6]. The most important dietary sources of iodine in industrialized countries are breads containing iodized salt and milk [2]. Iodide absorption in the gastrointestinal tract is mediated by the sodium-iodide symporter (NIS), which also mediates the uptake of iodide into the thyroid follicular cell (see Figure 1) [7,8]. Iodide is rapidly cleared from the circulation by the thyroid gland and kidneys. Thyroid clearance varies depending on iodine intake, from 10% of absorbed iodide in healthy individuals to more than 80% in chronic iodine deficiency [2].

### Iodide transport in thyroid cells

As illustrated in Figure 1, the NIS **(**SLC5A5**)**, a member of the solute carrier family 5, located at the basolateral plasma membrane of the thyroid follicular cells actively transports iodide into the thyroid using the electrochemical gradient generated by the Na,K-ATPase [9-11]. This process also requires a constitutive active potassium channel consisting of the KCNQ1 and KCNE2 subunits promoting potassium efflux [12-14]. Iodide efflux into the follicular lumen is mediated in part by pendrin, in conjunction with an as of yet unidentified channel. Pendrin (SLC26A4), a member of the multianion transporter solute carrier 26 family, is a coupled electroneutral iodide/chloride, iodide/bicarbonate, and chloride/bicarbonate exchanger [15-17]. At the intraluminal side, iodide is oxidized, a reaction that requires hydrogen peroxide (H_2_O_2_). The oxidation of iodide is mediated by thyroid peroxidase (TPO). TPO is also responsible for the iodination of selected tyrosil residues of thyroglobulin (organification), forming monoiodotyrosine (MIT) and diiodotyrosine (DIT) residues, and for the coupling of MIT and DIT resulting in the formation of T_3_ and T_4_[18]. The matrix for the synthesis and storage of T_4_ and T_3_ is thyroglobulin (Tg), a large glycoprotein secreted by the thyroid follicular cells [19,20]. H_2_O_2_ is generated by the dual oxidase 2 (DUOX2), a calcium dependent flavoprotein NADPH oxidase, which requires a maturation factor known as DUOXA2 [21]. T_3_ and T_4_ are released into the bloodstream, following micro- or macropinocytosis and lysosomal digestion of thyroglobulin by endopeptidases and exopeptidases [22-24]. Animal and cellular models suggest that the monocarboxylate channel (MCT8/SLC16A2) is involved in the efflux of thyroid hormones at the basolateral membrane [25,26]. MIT and DIT are deiodinated by the iodotyrosine dehalogenase, DEHAL1. This allows the re-utilization of iodide within the thyroid cell [27]. The molar ratio of secreted T4 to T3 is 11 to 1 due to intrathyroidal deiodination of T4 to T3 by type 1 and 2 deiodinases (D1 and D2) [28]. However, most T3 production occurs in extrathyroidal tissues and both, T3 and T4 can be converted to inactive forms via deiodination of the inner ring, by either type 3 deiodinases (D3) or D1 [29,30].

### Regulation of iodide transport

Iodide transport is dependent on the nutritional availability of iodide and on the stimulation of the thyroid stimulating hormone receptor (TSHR). Although the TSHR is constitutively active, it is susceptible to enhanced activation by TSH [31,32]. In addition, iodide uptake and organification are inhibited by high intracellular concentrations of iodide. Other factors have been shown to regulate iodide uptake, including thyroglobulin, cytokines, growth factors and estradiol.

1) **TSH**

TSH stimulates thyroid hormone synthesis and secretion. TSH is a glycoprotein with two subunits. The α subunit is identical to the glycoprotein hormones LH, FSH and hCG, whereas the β subunit is specific for the four hormones. TSH is synthesized and secreted in response to TSH releasing hormone (TRH) from the hypothalamus. Thyroid hormones negatively regulate the synthesis and secretion of both TRH and TSH. TSH stimulation of the G-protein coupled TSHR increases cAMP, which in turn, stimulates NIS transcription, half-life and subcellular distribution. TSH also upregulates the expression of TPO, Tg and the endocytosis of iodinated Tg [11] and increases the translocation of pendrin to the apical membrane of the thyroid follicular cell, thereby enhancing iodide efflux [33].

2) **Iodide**

Iodide is a major regulator of iodide accumulation and organification. Iodine intake has a negative effect on the expression of NIS and high doses of iodide block thyroid hormone synthesis via inhibition of organification (Wolff-Chaikoff effect) [34-37]. The adaptation to the initial inhibitory effect (the escape from the Wolff-Chaikoff effect) occurs as a result of decreased iodide transport. The escape is secondary to complex regulatory phenomena that involve, among others, decreased *NIS* gene transcription, increased NIS protein degradation and decreased NIS activity [38-40].

3) **Thyroglobulin (Tg)**

A role for Tg as an intrinsic regulator of iodide transport and thyroid hormone synthesis has been proposed to explain the heterogeneity of thyroid follicles and its differential expression of thyroid genes. Tg has been shown to decrease the gene expression of *NIS*, *TPO*, and *DUOX*[41-44].

4) **Cytokines and growth factors**

Cytokines such as TNF and interleukins inhibit iodide uptake and NIS expression. Insulin like growth factor 1 (IGF-1) affects thyroid hormone synthesis by downregulating the expression of NIS [10,45-47]. Transforming Growth Factor-β (TGF-β) has been shown to downregulate iodide transport by several mechanisms in different species, including inhibition of mRNA expression of TSHR, TPO, NIS, the Na, K-ATPase and thyroglobulin [48].

5) **Estradiol**

Estradiol downregulates the expression of NIS and iodide uptake in thyroid cells, possibly explaining the higher incidence of goiter in women. Estradiol also upregulates thyroglobulin [49,50].

### Thyroid conditions as they relate to iodide transport

The different mechanisms and disorders associated with abnormal iodide transport are summarized in Table 2. For detailed explanation, please refer to the text.

**Table 2:** Mechanisms and disorders associated with abnormal iodide transport

**Etiology**	**Conditions, drugs or environmental agents affecting this step in iodide transport**	**Manifestations**
Deficient nutritional iodine intake	Iodine deficiency disorders	**All ages:**
		• Goiter
		**Mother/fetus:**
		• Abortion
		• Stillbirth
		• Congenital anomalies
		• Perinatal mortality
		**Newborn:**
		• Infant mortality
		• Cretinism with neurological deficits and mental retardation
		**Child and adolescent:**
		• Growth retardation and delayed puberty
		**Child, adolescent and adult:**
		• Impaired mental function
		• Hypothyroidism
		• Increased risk to develop iodide induced-hyperthyroidism and toxic nodular goiter after exposure to iodine
Abnormal basal iodide uptake	NIS mutations (autosomal recessive)	Congenital hypothyroidism, typically with goiter. Iodide-trapping defect with little or no uptake of radioactive iodide both at the thyroid and salivary gland level
	Perchlorate, thiocyanate and nitrates	Increased risk of goiter development and hypothyroidism, specially in iodine deficient populations
	Goitrogens (soy and other flavonoids, glucosinolates and cyanogenic glucosides)	Increased risk of goiter development and hypothyroidism in iodine deficient populations
Apical iodide efflux	Pendred syndrome. Mutations in the *SLC26A4* gene (autosomal recessive)	Sensorineural hearing loss, variable phenotype of goiter and hypothyroidism and partial organification defect
	Congenital hypothyroidism with atrophic thyroid gland associated with SLC26A4 mutations (autosomal recessive)	Congenital hypothyroidism
Organification and coupling	*Tg* gene mutations (autosomal recessive)	Congenital hypothyroidism and/or variable degrees of goiter and hypothyroidism with low Tg levels
	*TPO* gene mutations (autosomal recessive)	Congenital hypothyroidism and/or variable degrees of goiter and hypothyroidism with partial or total organification defects
	Mutations in *DUOX2* or *DUOXA2* (autosomal recessive or dominant)	Transient or permanent congenital hypothyroidism
	Anti-thyroid medications (i.e. PTU, methimazole, carbimazole)	Medication-induced hypothyroidism
Recycling of iodide	Mutations in DEHAL1 (autosomal recessive)	Congenital hypothyroidism, goiter, increased MIT and DIT serum levels and severe urinary loss of MIT and DIT
Thyroid hormone degradation exceeds thyroid synthetic capacity	Overexpression of D3 in hemangiomas and gastrointestinal stromal tumors	Consumptive hypothyroidism with elevated rT3 and resistance to treatment with physiological doses of levothyroxine
Increased stimulation or constitutive activity of the TSHR or downstream pathways	TSHR stimulating immunoglobulins	Graves’ disease
		Transient congenital hyperthyroidism
	TSHR activating mutations	Sporadic congenital or autosomal dominant familial non-autoimmune hyperthyroidism (germline mutations)
		Toxic adenomas (somatic mutations)
	Pregnancy	hCG-induced gestational hyperthyroidism
	Somatic, activating mutations of G_sα_	Toxic nodular hyperthyroidism and hyperthyroidism in McCune Albright syndrome
Decreased stimulation or inactivation of the TSHR or downstream pathways	Presence of TSHR blocking immunoglobulins	Hypothyroidism
	Inactivating mutations of the TSHR (autosomal recessive)	Resistance to TSH with overt or compensated hypothyroidism
	Inactivating G_sα_ mutations	Hypothyroidism in the context of pseudohypoparathyroidism type Ia
Iodide mediated alterations in thyroid function	Iodine containing solutions	Transient hypothyroidism (Wolff-Chaikoff effect)
		In iodine deficiency: Hyperthyroidism (Jod-Basedow)
	Iodine containing contrast agents (iodine containing IV contrasts)	Transient hypothyroidism (Wolff-Chaikoff effect)
		In iodine deficiency: Hyperthyroidism (Jod-Basedow)
	Amiodarone	Amiodarone induced thyrotoxicosis (AIT): type 1: iodine inducedthyrotoxicosis, Jod-Basedow type 2: thyroiditis
		Amiodarone induced hypothyroidism (AMH); often associated with underlying autoimmune thyroid disease
Other defects in thyroid hormone release	Lithium	Hypothyroidism due to decrease release of T4

#### Disorders of iodine intake (DII)

Iodine deficiency causes hypothyroidism and goiter. Moreover, it is associated with an increased risk for abortion and stillbirths, congenital malformations, increased perinatal mortality, impaired growth and developmental retardation, impaired mental potential and decreased productivity. Iodine deficiency in critical periods of brain development and growth causes severe and permanent growth and cognitive impairment (cretinism) as thyroid hormones are required for myelination, neuronal differentiation and formation of neural processes in the cerebral cortex, the basal ganglia and the inner ear during the first trimester of gestation, and subsequently for brain growth and differentiation [11,51-58]. Importantly, pregnant women need higher amounts of iodide (Table 1). Even mild iodine deficiency during pregnancy may affect outcomes [54,59-61]. However, despite the efforts from the International Council for the Control of Iodine Deficiency Disorders (ICCIDD) to end a preventable form of hypothyroidism, goiter and mental retardation, thirty-two countries and about 246 million schoolchildren are estimated to have insufficient iodine intake [4,5]. In the US, the median urinary iodine concentration decreased by over 50% between the early 1970s and the early 1990s and even though most of the US population remains iodine sufficient, the aggregate data from NHANES 2007–2010 indicates that a subset of young women and pregnant women may have mild iodine deficiency [3]. Popular foods among young women, marketed for weight loss, are deficient in iodine [62]. Furthermore, prenatal vitamins have inconsistent amounts of iodide content [63,64]. Iodine supplementation is recommended not only for pregnancy, but also during lactation [65] as iodine supplementation given to a lactating mother provides adequate iodine to their infants [66]. Criteria for assessing iodine nutrition in populations based on school age children and in pregnant and lactating women are summarized in Table 3[2,4,58]. Thyroglobulin is also a sensitive method to assess iodine intake [67,68]. Disorders of iodide transport (see below) are influenced by iodine intake. In addition, other questions remain, such as whether mild, transient congenital and/or subclinical hypothyroidism could be impacted by improving iodine intake.

**Table 3:** **Epidemiological criteria for assessing iodine nutrition based on median iodine urine concentration in school age children and median iodine concentration in pregnant women**[1]

**Population**	**Median urinary iodine (μg/L)**	**Iodine intake**
**School age children (older than 6 years old)**	<20	Insufficient (severe)
	20-49	Insufficient (moderate)
	50-99	Insufficient (mild)
	100-199	Adequate
	200-299	Above requirement
	<300	Excessive
Pregnant and lactating women	<150	Insufficient
	150-249	Adequate
	250-499	Above requirements
	<500	Excessive

#### Disorders of iodide transport

1) **Disorders associated with abnormal basolateral uptake**

 Mutations in the *NIS* gene

Homozygous or compound heterozygous inactivating mutations of the *NIS* can cause congenital hypothyroidism. The thyroid may be normal at birth, but enlarges overtime due to TSH stimulation, unless thyroid hormone replacement is started. Affected individuals have an iodide-trapping defect with little or no uptake of radioactive iodide both in the thyroid and the salivary glands [69].

2) **Disorders associated with abnormal apical iodide efflux**

2.2) Congenital hypothyroidism with hypoplastic thyroid gland due to *PDS/SLC26A4* mutations

Kühnen et al. [72] found biallelic mutations in the *SLC26A4* gene in two individuals from two families with hypoplastic thyroid glands. They speculated that the hypoplasia may be caused by “secondary atrophy”. However, the described mutations have also been reported in patients with Pendred syndrome, while the patients described in this study had thyroid hypoplasia. One case had apparently a normal hearing test. Nevertheless, imaging studies of the inner ear were not obtained. A second patient had deafness and mental retardation. The authors did not comment of the hearing function of the other four patients with hypoplastic thyroid glands harboring mutations on the *SLAC26A4* gene. Moreover, the thyroid volumes of the index patients early in life are unknown [72]. Hence, it is not clear if patients presenting with hypoplastic thyroid glands may be within the spectrum of Pendred syndrome or not, and the mechanism causing thyroid atrophy needs to be further elucidated; it could, e.g., involve destruction of thyroid cells by the retained misfolded proteins [17].

2.1) Pendred syndrome

Pendred syndrome is an autosomal recessive disorder caused by mutations in the *PDS/SLC26A4* gene. It is characterized by sensorineural hearing loss associated with malformations of the inner ear (enlarged vestibular system), variable degrees of goiter and hypothyroidism and a partial iodine organification defect diagnosed by the perchlorate discharge test (see below) [17,70,71].

3) **Disorders of organification and coupling**

3.3) Dual oxidases and its chaperones (DUOX2/DUOXA2)

DUOX1 and DUOX 2 are NADPH flavoproteins that share 83% sequence similarity. Both *DUOX* genes are expressed in the thyroid but their expression is not restricted to the thyroid. The *DUOX2* and *DUOXA2* genes are contiguous (together with their homologues *DUOX1* and *DUOXA1*) on the long arm of chromosome 15. Only mutations in DUOX2 and in DUOXA2 have been found to cause congenital hypothyroidism [21,76-78]. In some cases, transient hypothyroidism occurs. This was initially postulated to be secondary to heterozygous mutations, while biallelic *DUOX2* mutations were thought to cause permanent hypothyroidism. However, transient hypothyroidism also occurs in individuals with biallelic mutations [77]. The role of DUOX1 in compensating for the loss of DUOX2 is unclear at this time and it is thought that iodide availability may also affect the phenotype.

3.2) Thyroid peroxidase (TPO)

Recessive TPO defects are among the most common causes of congenital hypothyroidism secondary to dyshormonogenesis. Patients may have a partial or total organification defect. A recent study in the Netherlands found that *TPO* gene defects are the most common cause of a total organification defect, as diagnosed by a positive perchlorate test with a discharge of < 90% [75].

3.1) Thyroglobulin (Tg)

Biallelic mutations in the *Tg* gene can cause congenital hypothyroidism. The clinical spectrum ranges from normal thyroid function to overt hypothyroidism. The majority of patients have congenital goiter or develop goiter shortly after birth. The serum Tg concentrations are very low. Affected individuals are homozygous or compound heterozygous for inactivating mutations. Defective Tg molecules are typically retained in the ER and routed for degradation. However, some truncated proteins can be secreted and are sufficient for partial thyroid hormone synthesis [19,73,74].

4) **Disorder of intra-thyroidal iodide recycling**

4.1) Dehalogenase (DEHAL)

Mutations in the *DEHAL1* gene (*IYD*) can cause congenital hypothyroidism, goiter, increased MIT and DIT serum levels and urinary loss of MIT and DIT [27,79,80]. Variable mental deficits can occur, depending on age of diagnosis and on whether hypothyroidism occurs during development [11,79].

#### Disorders of abnormal iodide transport regulation

1) **Conditions affecting TSH signaling**

1.1) Hyperthyroidism

Conditions causing overstimulation of the TSHR increase iodide uptake and thyroid hormone synthesis. In Graves’ disease, the production of TSHR-stimulating immunoglobulins causes increased thyroid cell proliferation, iodide uptake and thyroid hormone synthesis. These IgG antibodies can cross the placenta and are the most common cause of congenital hyperthyroidism [31,32,81]. Rarely, activating mutations of the TSHR are the cause of excessive iodide uptake and hyperthyroidism. They can present as somatic mutations in thyrotoxic adenomas, as autosomal dominant familial non-autoimmune hyperthyroidism, or as sporadic *de novo* germline mutations [31]. Activating mutations in the downstream G protein G_sα_ can also cause non-autoimmune hyperthyroidism; this occurs through somatic mosaicism affecting thyroid cells in McCune Albright syndrome, or as isolated activating mutations in toxic adenomas [82,83]. During pregnancy, hCG stimulates iodide transport and thyroid hormone synthesis through stimulation of the TSHR. hCG has structural similarity to TSH and leads to a transient increase in thyroid hormone synthesis, resulting in lower TSH levels. In some women, the high hCG levels can cause overt hyperthyroidism and be associated with hyperemesis gravidarum. hCG-secreting trophoblastic tumors (hydatidiform mole, choriocarcinoma) are rare causes of hyperthyroidism [84].

2) **Iodine-induced conditions**

Medications or environmental agents can affect the concentration of intracellular iodide or its regulatory mechanisms. Amiodarone is an antiarrhytmic drug that contains two atoms of iodine in an inner benzene ring, similar to thyroid hormones. Each 200 mg tablet of amiodarone contains 75,000 μg of iodine [92]. It can cause amiodarone-induced thyrotoxicosis (AIT) via two different mechanisms. AIT type 1, which occurs more frequently in iodine deficient areas, is caused by excessive thyroid hormone synthesis by nodular thyroid tissue that has lost its autoregulatory capacity (Jod-Basedow phenomenon; Jod = iodine in German; Karl von Basedow = German physician who described thyrotoxicosis associated with exophthalmos and goiter) [93-97]. The Jod-Basedow effect can be caused by any form of iodine excess such as contrast agents or iodine-containing solutions [98-101]. Currently used, water soluble iodinated contrast agents provide exposure to about 13,500 μg of free iodine per computerized tomography (CT) imaging study [92]. AIT type 2 occurs secondary to amiodarone-induced thyroiditis. Amiodarone can also cause hypothyroidism (AIH), particularly in patients with underlying autoimmune thyroid disease. Lithium is another widely used drug known to affect thyroid function. Among other effects, it appears to promote iodide retention in the thyroid and it decreases the release of thyroid hormone from the gland [102-104]. Other effects of amiodarone and lithium are reviewed elsewhere [93-96,102-105].

1.2) Hypothyroidism

Conditions causing a decreased or absent response of the TSHR to TSH cause inadequate iodide uptake and thyroid hormone synthesis. Autoimmune hypothyroidism can be caused by the presence of blocking thyrotropin binding inhibitor immunoglobulins (TBII). These antibodies cross the placenta and may cause transient congenital hypothyroidism [85,86]. Resistance to TSH can be caused by molecular defects affecting the transmission of the TSH stimulatory signal, most commonly due to biallelic loss of function mutations of the TSHR. The phenotypes vary from a hypoplastic thyroid gland with severe congenital hypothyroidism to mild hyperthyrotropinemia with an euthyroid state [87,88]. Inactivating mutations in the G_sα_ cause mild hypothyroidism, such as seen in pseudohypoparathyroidism [89-91].

#### Consumptive hypothyroidism

Hemangiomas and gastrointestinal stromal tumors may express high levels of D3. This enzyme catalyzes the conversion of T4 to rT3 and of T3 to T2, i.e. inactive forms of thyroid hormone. This causes a unique form of hypothyroidism due to increased degradation of thyroid hormones at a rate that exceeds the synthetic capacity of the stimulated thyroid gland [106-108]. These patients have significantly elevated rT3 levels and require unusually large doses of levothyroxine in order to compensate for the increased degradation of T4 and T3.

#### Drugs, diet and environmental agents affecting iodide transport and metabolism

1) **Perchlorate, thiocyanate and other environmental agents**

In addition to its iodide transport activity, NIS also transports other anions [11,109], including selenocyanate (SeCN^−^), thiocyanate (SCN^−^), chlorate (ClO3^−^), and nitrate (NO_3_^−^). Pertechnetate (TcO_4_), perrhenate (ReO_4_^−^) and perchlorate (ClO_4_^−^) are also NIS substrates [11]. Perchlorate is a competitive NIS inhibitor. Perchlorate salts are used as oxidizers in solid propellants for a wide range of uses; perchlorate is not biodegradable and it is found in drinking water, food and multivitamins [110,111]. The Environmental Protection Agency (EPA) established a minimum reporting level (MRL) of 4 μg/L [112]. Perchlorate can be transported by NIS into the thyroid and the mammary gland, which would potentially decrease iodide supply in the breast milk and affect the newborn’s iodide uptake by the thyroid gland [113]. Kirk et al. found an inverse correlation between breast milk iodine and perchlorate concentration [114]. However, other studies do not show a similar correlation [115,116]. In healthy adults, exposure to perchlorate for 6 months with doses as high as 3 mg/day did not affect thyroid function [117] and thus, the consequences of environmental perchlorate exposure still remain controversial [111]. Thiocyanate is a less potent inhibitor of NIS-mediated iodide transport than perchlorate. Exposure to thiocyanate comes mainly from cigarette smoke (containing cyanide, which is metabolized to thiocyanate) and from the diet (see below). Smoking seems to affect iodide secretion into the breast milk [118]. The available studies trying to address the effect of smoking on thyroid function are not conclusive. It appears that smoking is associated with goiter and hypothyroidism in iodine deficient regions, whereas smokers have lower TSH levels in iodine sufficient areas [119,120]. Although the risks of perchlorate and thiocyanate exposure in healthy adults remain unresolved, a recent study indicates that a combination of perchlorate and thiocyanate exposure with low iodine intake lowers free thyroxine concentration by about 12% [121]. Nitrates are widely present in soils and water and come from natural decomposition of organic materials. Sodium nitrite is also used as a preservative. The average intake of nitrates in adults is 75–100 mg/day and 80% comes from vegetables. Vegetarians may ingest 2.5 times the average intake. High ingestion of nitrates usually comes from contaminated water. The EPA defined the maximum contaminant level at 10 mg/L or 10 ppm [112]. Exposure to high levels of nitrates due to polluted water has been shown to cause thyroid dysfunction and goiter [122,123].

2) **Medications used to treat hyperthyroidism**

The anti-thyroid drugs used in the US include propyl-thiouracil (6-propyl-2-thiouracil) and methimazole (1-methyl-2-mercaptoimidazole). Carbimazole, which is metabolized to methimazole, is widely used in other parts of the world. These thionamide drugs are actively concentrated in the thyroid and their primary effect consists in inhibiting the TPO-mediated organification [124].

3) **Diet**

Cruciferous vegetables like cabbage, kale, broccoli, turnips and cauliflower contain glucosinolates. Cassava (linamarin), lima beans, sweet potatoes, sorghum and flaxseed contain cyanogenic glucosides. Both, glucosinolates and cyanogenic glucosides are metabolized to thiocyanate that competes for thyroid iodide uptake. These substances can aggravate iodine deficiency and contribute to goiter development. Hence, they are called goitrogens. Soy and millet contains flavonoids that may inhibit TPO activity. Use of soy-based formula without added iodide can produce hypothyroidism and goiter in healthy infants [125-128].

#### Iodine as a tool for diagnosis and treatment of thyroid disorders

The ability of the thyroid to concentrate iodide is widely used in the diagnosis and treatment of thyroid disorders. Commonly used diagnostic tests such as the radioactive iodine uptake and (whole body) scan rely on the ability of thyroid tissue to concentrate radioactive labeled iodine. I^−131^, I^−123^ and I^−124^ (a positron emission tomography (PET) tracer) are the major radionuclide agents used for the diagnosis of thyroid diseases (Table 4). These tests can be used to differentiate a hyperactive thyroid, with increased uptake (e.g. Graves’ disease, toxic nodules), from an underactive thyroid with decreased iodine uptake, secondary to either thyroid damage or inactivation (e.g. thyroiditis, factitious thyrotoxicosis) or a blockade in thyroid uptake (e.g. mutation in NIS). Whole body scans with radioactive iodine are useful for the staging and planning of therapy of well-differentiated thyroid cancer [129]. Because of the ability of NIS to transport pertechnetate (TcO_4_^−^), ^99m^TcO_4_^−^, an isotope with no β emission and a short half-life, can be used to image thyroid tissue (see Table 3) [130-132]. The perchlorate (ClO_4_^−^) discharge test is a functional test that uses ClO_4_^−^ to inhibit NIS and radioactive iodine to diagnose partial or total organification defects. This test relies on the fact that iodide transported into the thyroid is covalently bound to Tg (organification). Radioactive iodide is administered, followed by radioactive uptake measurement in the neck using a gamma camera. Two hours later, uptake is blocked using the competitive NIS inhibitor ClO_4_^−^ and the radioisotope counts are measured again over the next hour. Organified iodine is retained, while free, unbound iodide is washed out. A test is considered positive if <10% of activity is discharged after ClO_4_^−^ administration. Partial organification defects show a 10-90% discharge, while discharge <90% is consistent with total organification defect [19,21,133-135].

**Table 4:** **Radionuclides used for evaluation and management of thyroid disorders**[132]

**Radionuclide**	Radioactive emissions (keV*)	**Half-life**	**Clinical use**
^**123**^**I**	γ 159 keV	13.2 hours	Thyroid and whole body scanning
^**131**^**I**	γ 364 keV	8.09 days	Thyroid and whole body scanning
	β 637 keV		Treatment of Graves’ disease, toxic adenomas, thyroid cancer
^**124**^**I**	β^+^ (positron emitter) γ 603 keV	4.2 days	Whole body scanning Dosimetry
^**99m**^**T**_**c**_**O**_**4**_	γ 140 keV	6 hours	Thyroid scanning

*keV = kiloelectron volt (1.60217657 × 10^−16^ joules)

#### Iodine in the prevention of thyroid disorders and public health

Potassium iodide and potassium perchlorate can be used to protect the thyroid from exposure to I-131 after accidental release from nuclear plant reactors to prevent hypothyroidism and thyroid cancer [136].

#### New developments in iodide transport in the diagnosis and management of thyroid cancer

Poorly differentiated thyroid cancer cells show decreased or absent iodide uptake. This is associated with decreased expression or membrane insertion of NIS at the plasma membrane. For this, reason, there is a great interest in re-differentiating agents that increase NIS expression and membrane insertion [11]. For example, selumetinib, a MAPK (MEK1/MEK2) inhibitor can result in improved radioactive iodine uptake and retention in some patients with radioiodide resistant thyroid cancer [137].

#### Applications of iodide transport outside the thyroid

Outside the thyroid, non-regulated iodide accumulation, without organification, is known to occur in the lactating mammary gland, salivary and parotid glands, gastric mucosa, small intestine, choroid plexus and the ciliary body of the eye [11,46]. In addition, NIS is expressed in other tissues [138], however, the physiological relevance of NIS in these tissues in unclear, except in the lung, where oxidation of iodide improves anti-viral defenses [11,139]. Endogenous NIS expression occurs in breast cancer and cholangiocarcinoma. Currently, ongoing research is exploring the use of ^131^I^−^ to treat these types of cancers. The fact that NIS transports perrhenate defines ^188^ReO_4_^−^ as a candidate to increase radiation dose delivery to these tumors [11]. Transduction of viral vectors containing the cDNA of *NIS* under the control of heterologous promoters (e.g. the *PSA* promoter) are used experimentally in order to treat other malignancies (such as prostate cancer) [140].

## Conclusions

In conclusion, iodide transport is of essential physiological importance for thyroid hormone synthesis. The understanding of iodide transport and its regulation has been fundamental in characterizing the spectrum of thyroid disorders. The ability of thyroid follicular cells to concentrate iodide can be used for diagnostic and therapeutic purposes and the elucidation of the molecular events governing iodide uptake also has important implications because it allows to target NIS for re-differentiation therapies and to use it in non-thyroidal tissues.

## Abbreviations

D1: Type 1 deiodinase; D2: Type 2 deiodinase; D3: Type 3 deiodinase; DIT: Diiodotyrosine; DUOX: Dual oxidase; DEHAL1: Dehalogenase; H_2_O_2_: Hydrogen peroxide; ICCIDD: International Council for the Control of Iodine Deficiency Disorders; MIT: Monoiodotyrosine; PDS: Pendrin; NIS: Sodium iodide symporter; Tg: Thyroglobulin; T3: Triiodothyronine; T4: Thyroxine; TPO: Thyroid peroxidase; TRH: TSH releasing hormone; TSH: Thyroid Stimulating Hormone; TSHR: TSH-receptor; WHO: World Health Organization; US: United States.

## Competing interests

The authors declare that they have no competing interests.

## Authors’ contributions

LP made significant contributions to the conception, planning, review of literature, writing, reviewing and editing the manuscript. PK made significant contributions to reviewing content, editing and approving the final version of the manuscript. Both authors read and approved the final manuscript.

## Author’s information

LP is a Clinical Assistant Professor of Pediatric Endocrinology with interest in pediatric thyroid disorders and thyroid physiology. PK is an Associate Professor of Endocrinology and he is the director ad interim of the Center of Genetic Medicine at Northwestern University. His clinical focus is directed towards thyroid dysfunction and thyroid cancer. His research interests include genetic endocrine disorders, in particular of the thyroid and the pituitary gland.
